# Can music with prosocial lyrics heal the working world? A field intervention in a call center

**DOI:** 10.1111/jasp.12282

**Published:** 2014-07-23

**Authors:** Karen Niven

**Affiliations:** Manchester Business School, University of Manchester

## Abstract

Music with lyrics about helping is shown to reduce aggression in the laboratory. This paper tests whether the prosocial lyric effect generalizes to reducing customer aggression in the workplace. A field experiment involved changing the hold music played to customers of a call center. The results of a 3 week study suggested that music significantly affected customers, but not in the way suggested by previous laboratory experiments; compared with days when instrumental background music was played, caller anger and employee exhaustion were lower on days when callers were played popular music with neutral, but not prosocial, lyrics. The findings suggest that music influences customer aggression, but that the prosocial lyric effect may not generalize from the laboratory to the call center.

Most customer service organizations do not pay a great deal of attention to the music they play to their customers, with many opting to play instrumental background music to avoid licensing fees. However, a recent body of laboratory-based research from social psychology has suggested that lyrics can influence people's interpersonal behavior in desirable ways; specifically, listening to music with lyrics about helping (termed “prosocial” lyrics) reduces people's aggression (Greitemeyer, 2011). This has important implications for the workplace, because customer aggression is a real problem that creates substantial costs for customer service organizations. In call centers, for example, employees deal with an average of 10 angry callers per shift, leaving them feeling emotionally drained and impeding their efficiency (Grandey, Dickter, & Sin, 2004; Rafaeli et al., 2012). Music with prosocial lyrics could therefore serve as a low-cost intervention for such organizations, with a huge potential payoff in terms of staff well-being and performance. The current study presents the first test of whether the effect of prosocial lyrics on aggression generalizes beyond the laboratory, with a field experiment conducted in a customer service call center.

## Music and interpersonal behavior

It is well-established that music influences people's interpersonal behavior. For example, Fried and Berkowitz's (1979) laboratory experiment showed that participants who listened to soothing (classical) music reported that they were willing to devote more time to helping an experimenter with further research studies, compared with participants who listened to aversive (jazz) music or no music. In a more recent laboratory study, Krahé and Bieneck (2012, Study 1) found that participants exposed to pleasant (classical) music reported more positive mood than those exposed to aversive (hard core and techno) music or no music. In turn, participants' positive mood was negatively related to their feelings of anger toward a “co-participant” who provided them with evaluative feedback, and the negativity of their feedback to the “co-participant,” which was viewed as a measure of aggressive behavior. Outside the laboratory, North, Tarrant, and Hargreaves (2004) played 646 gym members either uplifting (up-tempo pop) or annoying (avant-garde computer) music while they worked out. After their workout, those in the uplifting music condition agreed to distribute significantly more leaflets for a sporting charity.

The effect of music on interpersonal behavior can be explained with reference to the general learning model of social behavior (Buckley & Anderson, 2006), which proposes that the content of media, such as music, affects people's affect and arousal, readying the person to act. Negative, high arousal states, such as anger can lead to a “fight” reaction characterized by aggression, whereas positive emotion states are associated with cooperation and helping responses. Similarly, the general aggression model (Anderson & Bushman, 2002) states that stimuli such as music influence a person's internal (cognitive and affective) state and subsequent appraisal and decision processes, making particular interpersonal behaviors more or less likely to occur. Thus, whereas music experienced as aversive should lead to negative affect and so inhibit altruism and encourage aggression, music experienced as pleasant should elicit positive affect and so promote altruism and reduce aggression.

Although the above studies focus on music genre as the primary feature that affects how we act toward others, the semantic content of music may have a particularly important role to play in influencing interpersonal behavior. Research suggests that exposure to emotive (e.g., violent) words can shape people's feelings and actions, even when the words have not been consciously recognized (Bargh, Chen, & Burrows, 1996). Similarly, the lyrics of music could influence people's affect and arousal and in turn their interpersonal behavior. Across five studies Anderson, Carnagey, and Eubanks (2003) provide support for this notion, reporting that participants who were played songs with violent lyrics felt more hostile and had more aggressive thoughts compared with those played similar songs that had nonviolent lyrics. Fischer and Greitemeyer (2006) further showed that compared with an equivalent sample who listened to music with neutral lyrics, men exposed to music with misogynistic lyrics behaved more aggressively to a person they believed to be a fellow participant (e.g., by administering more hot chili sauce), especially when the fellow participant was female.

Although the above literature indicates that the lyrics of music can negatively affect interpersonal behavior, a recent program of experimental laboratory-based research by Tobias Greitemeyer provides compelling evidence that the lyrics of music can also have a positive influence on interpersonal behavior (Greitemeyer, 2009a, 2009b, 2011). Greitemeyer proposed that exposure to songs with “prosocial” lyrics about helping others (e.g., Michael Jackson's “Heal the world,” which has lyrics such as “Heal the world. Make it a better place, for you and for me and the entire human race”) would improve people's interpersonal behavior. In his studies, student participants are randomly assigned to listen to either songs with prosocial lyrics or songs with non-prosocial (“neutral”) lyrics. The songs in both conditions are matched so that they come from the same artists, and in rigorous pilot testing Greitemeyer (2009a) confirmed that the songs differed primarily in terms of prosocial lyrical content and not in terms of participants' liking or arousal. In a series of seven studies published across two papers, Greitemeyer (2009a, 2009b) demonstrated that compared with songs with neutral lyrics, listening to songs with prosocial lyrics increased empathic feelings and helping behaviors, such as donating money to charity and helping an experimenter to pick up pencils that had been dropped. In a further series of five studies, Greitemeyer (2011) demonstrated that music with prosocial lyrics could reduce aggressive feelings and behaviors, such as negatively evaluating a doctoral candidate.

Although the results of Greitemeyer's (2011) research are promising, they have yet to be replicated in the field. Many researchers maintain that people's experiences of music are situationally dependent. For example, Ruud (2010) argues that “what is aesthetically significant in music cannot be decided from disembodied analysis; it must take into account the particular situation and the particulars in the music” (p. 56). Music might therefore be experienced differently in a field context, in which people who are played music are likely to have goals and emotions that are important and relevant to them, compared with the highly controlled, neutral laboratory environment studied by Greitemeyer. Only one published study has examined the effect of music with prosocial lyrics in the field, reporting a link between prosocial lyrics and patrons' tipping behavior in a restaurant (Jacob, Guéguen, & Boulbry, 2010). Thus, it is still unknown whether the effect of prosocial lyrics on aggression generalizes to the field.

## Music with prosocial lyrics as an intervention for customer aggression

Customer service represents an important field context that stands to benefit from music with prosocial lyrics, due to the relatively high prevalence of anger directed toward employees by customers in service organizations (Grandey, Kern, & Frone, 2007). Unsurprisingly, dealing with aggressive customers takes a substantial toll on employees; in their meta-analysis, Hershcovis and Barling (2010) reported sizable effects of customer aggression on employees' emotional exhaustion (.36), psychological distress (.22), and physical health (−.19). Not only might employees' well-being be compromised by their exposure to customer aggression, so too might their performance. Laboratory simulations of workplaces have provided evidence that dealing with verbally aggressive customers reduces participants' recall, recognition, and working memory, as well as the quality of task performance, because it depletes the resources available to devote to cognitive tasks (Rafaeli et al., 2012). A field study in a call center further found that displays of negative emotion from customers led to employees' experiences of negative affect and in turn to reduced productivity (e.g., in terms of numbers of calls taken per hour; Rothbard & Wilk, 2011). Thus, there is a clear need for interventions to tackle customer aggression. However, although some interventions have been tested for dealing with aggression from people inside the organization (Hoel & Giga, 2006), dealing with aggressive customers, whose behavior is not directly under the control of the organization, remains a significant challenge.

The present research addresses this challenge by applying what is known from laboratory research to create a novel intervention for customer aggression. Specifically, the research tests whether prosocial music can be used as an intervention to reduce caller aggression in a customer service call center. A call center provides an excellent environment to test prosocial music for two main reasons. First, callers are frequently angry, as the purpose of many calls is to complain about a product or service (Grandey et al., 2004), meaning that an intervention could potentially be very beneficial. Second, customers in this context already listen to music while waiting for their call to be answered (which can even act as a further source of annoyance; Unzicker, 1999), and prior research has suggested that callers' experiences of hold music can have a strong influence on their behavior (e.g., influencing the length of time callers are willing to wait for their call to be answered; North, Hargreaves, & McKendrick, 1999).

A field experiment was conducted, with the intervention administered by changing the music played to callers while they waited for their calls to be answered. Following Greitemeyer (2011), music with prosocial lyrics was contrasted to music by the same artists that had neutral lyrics. In the present study, music with prosocial lyrics was also contrasted to the music that was already played in the call center (instrumental background music), to test whether the intervention represented an improvement over the current music used. Based on Greitemeyer (2011), it was expected that compared with days when neutral or instrumental is played, caller anger and employee exhaustion would be lower, and employee performance would be better, on days when callers were played music with prosocial lyrics.

## Method

### Participants

The sample comprised customer service representatives working for a branch of an organization that provides outsourced call center services to businesses in the United Kingdom. Staff members were asked to sign up to a study ostensibly about the effects of caller aggression. Of the 31 staff members employed, 25 elected to take part in the research, 15 of whom were full-time workers, and 10 of whom worked variable time (19 females and 6 males; *M*_age_ = 33.67 years, standard deviation [*SD*] = 13.02; *M*_tenure_ = 3.74 years, *SD* = 4.69). Participants were given a £40 (approximately $60) incentive for their time.

### Design

A repeated measures design was used with three conditions (prosocial, neutral, no change) that differed depending on the music played to callers while waiting for their calls to be answered. Over a 3 week period, the music was changed daily such that there were 7 days of each music type, with each day of the week included in each condition to balance out any potential day-of-week effects in caller anger. Employees and callers were not informed of the intervention and so were blind to the conditions. A diary methodology was used to collect the dependent variables, whereby data were collected hourly and daily from employees and from objective call center records. Across the whole study period, participants completed a total of 1937 hourly ratings (87% compliance rate) and 258 daily ratings (92% compliance rate), and there were 279 observations of objective performance. There were no significant differences between participants who fully complied with the study protocol (i.e., those who provided data on every possible opportunity) and those who did not, in any of the main study variables (*p*s > .05).

### Intervention

For the prosocial and neutral conditions, all tracks were taken from Greitemeyer's (2009a, 2009b, 2011) research, which has demonstrated that the songs chosen differ significantly in terms of prosocial lyrical content but not in terms of liking or arousal. The songs in the prosocial condition were Michael Jackson's “Heal the world,” The Beatles “Help!”, and Bob Sinclar's “Love generation.” Two minute samples of these songs featuring the key prosocial lyrics were selected and made into a single track that was played on a loop while customers waited for their calls to be answered. The songs in the neutral condition were Michael Jackson's “On the line,” The Beatles “Octopus's garden,” and Bob Sinclar's “Rock this party,” also cut in length and played on a loop. For the no change condition, the music usually used in the call center (instrumental background music) was played to callers while on hold. In order to maintain ecological validity in testing whether music with prosocial lyrics represented an improvement over the current situation at the call center, this instrumental music was left unchanged and was not cut in the same way as the music used in the other two conditions.

### Measures

#### Caller anger

Caller anger was rated by employees at the end of each working hour for the 3 week study period, using five items from the UWIST mood checklist (Matthews, Jones, & Chamberlain, 1990; e.g., “Annoyed”; α = .96 for all observations), rated on a 1 (*not at all*) to 5 (*a great deal*) scale. Hourly measures were used to reduce retrospective recall biases regarding aggressive events during the day (Hershcovis & Reich, 2013).

#### Employee exhaustion

Employees' current feelings of emotional exhaustion were measured at the end of each working day during the study period, using the four highest loading items from the emotional exhaustion subscale of Maslach and Jackson's (1981) burnout scale (e.g., “I feel emotionally drained”; α = .86 for all observations), again rated on a 1 (*not at all*) to 5 (*a great deal*) scale.

#### Employee performance

Two indicators of employees' daily performance were collected using objective performance measures provided by the organization: number of calls taken and mean call length. Both measures are common metrics used in customer service call centers, with good performance represented by efficiency, i.e., a high number of calls taken and a low mean call length.

#### Call hold time

The mean length of time that callers spent on hold was calculated using objective call records, to control for potential differences in terms of how long callers were able to listen to each type of music, and busyness of the call center (which could have implications for caller anger and employee exhaustion).

### Analyses

The data were analyzed using multilevel modeling, as hours were nested within days, which in turn were nested within persons. Gender, age, tenure, and mean caller hold time were all entered as control variables. Experimental condition was included as a categorical predictor variable. Intercepts were allowed to vary, as were slopes for the predictor variable to allow for random as well as fixed effects of the intervention. A three-level multilevel model was run to test effects of the intervention on caller anger (measured hourly), while two-level models were run to test effects on employee exhaustion and performance (measured daily).

## Results

Means, standard deviations, and raw correlations between the main study variables are displayed in Table 1. As can be seen, caller anger was strongly related to employees' emotional exhaustion, but not to employee performance.

**Table 1:** Means, Standard Deviations, and Raw Correlations between Study Variables

	*Mean*	*SD*	1	2	3	4	5	6	7
1. Gender	1.76	0.44	—						
2. Age	33.67	13.02	.26	—					
3. Tenure	3.74	4.69	.12	.48*	—				
4. Caller anger	1.61	0.76	−.38†	−.49†	−.21	—			
5. Employee exhaustion	1.25	0.52	−.40†	−.25	−.27	.27**	—		
6. Mean hold time	74.78	87.80	.10	−.24	−.26	.09	.16*	—	
7. Number of calls	65.48	31.72	.23	.15	−.22	.08	−.06	−.01	—
8. Mean call length	139.32	33.96	.04	.02	−.11	.07	.11†	.50**	−.20**

*Note.* Analyses are conducted at the individual level (with day- and hour-level data aggregated) for correlations involving the individual variables of gender, age, and tenure (*n* = 25), and at the day level (with hour-level data aggregated) for all other analyses (*n* = 258). Gender is coded 1 = male, 2 = female. *SD* = standard deviation. **p < .01. *p < .05. †*p* < .1.

The results of the multilevel modeling analyses (see Table 2) revealed a significant effect of condition on caller anger, *F*(2, 12.37) = 4.00, *p* < .05. However, estimates of fixed effects revealed that the significant difference was between the neutral (estimated marginal mean = 1.52) and no change (estimated marginal mean = 1.70) conditions (fixed estimate = .19, standard error [*SE*] = .07, *p* < .05). There were no significant differences in caller anger between the prosocial condition (estimated marginal mean = 1.60) and either the no change condition or the neutral condition. The results for employee emotional exhaustion followed the same pattern. The overall effect of condition on employee emotional exhaustion was significant, *F*(2, 6.01) = 4.10, *p* < .05, and estimates of fixed effects revealed a significant difference between the neutral (estimated marginal mean = 1.14) and no change (estimated marginal mean = 1.31) conditions (fixed estimate = .17, *SE* = .08, *p* < .05). There was no significant difference between the prosocial condition (estimated marginal mean = 1.21) and the no change condition, and exhaustion was marginally lower in the neutral condition than the prosocial condition (fixed estimate = −.07, *SE* = .04, *p* = .07). Thus, in both analyses, the main differences were between the neutral and the no change conditions, with lower caller anger and lower employee emotional exhaustion on days when neutral music was played. There were no effects of condition on the number, *F*(2, 19.41) = 1.75, *p* = .20, or mean length, *F*(2, 20.93) = 1.72, *p* = .20, of calls taken by employees. The predictions of the study were therefore not supported.

**Table 2:** Effects of the Music Intervention on Caller Anger and Employee Exhaustion

Model	−2*LL	Predictor variables	*F* value	Fixed estimate	Fixed effects *SE*
1) Effects on caller anger	3938.51	Intercept	439.07**	1.60**	.09
		Gender	0.98	0.06	.06
		Age	10.35**	−0.01**	.01
		Tenure	0.17	<0.01	<.01
		Mean caller hold time	0.62	<0.01	<.01
		Condition	4.00*		
		Prosocial versus no change		0.10	.07
		Prosocial versus neutral		−0.08	.07
		Neutral versus no change		0.19*	.07
2) Effects on employee emotional exhaustion	2262.84	Intercept	63.60**	1.21**	.18
		Gender	1.99	0.20	.14
		Age	<0.01	<0.01	<.01
		Tenure	0.26	−0.01	.01
		Mean caller hold time	16.08**	<0.01**	<.01
		Condition	4.10*		
		Prosocial versus no change		0.10	.08
		Prosocial versus neutral		−0.09†	.04
		Neutral versus no change		0.17*	.08

*Note.* LL = log likelihood; *SE* = standard error. Gender is coded 1 = male, 2 = female. **p < .01. *p < .05. †*p* < .1.

## Discussion

Although music with prosocial lyrics has been shown to reduce listeners' aggressive feelings and actions in the laboratory (Greitemeyer, 2011), in a customer service call center exposure to prosocial lyrics did not show the samebenefits. Callers who were played music with lyrics about helping (e.g., Michael Jackson's “Heal the world”) were not rated as less angry than those who were played instrumental music, nor than those who were played music with neutral lyrics. Consistent with this, playing music with prosocial lyrics to callers also did not have positive consequences for employees' emotional exhaustion or their performance. Unexpectedly, however, music with neutral lyrics did have a positive effect compared with the instrumental music usually played to callers, leading to reduced caller anger and employee emotional exhaustion. Given that employees and callers were unaware that an intervention was occurring and that the two dependent measures were assessed at different times of the day (one hourly, one at the end of the day), these findings are unlikely to be due to expectations or a common method effect.

There are several reasons why music with prosocial lyrics may have beneficial effects on anger in the laboratory but not in a call center. Unlike the laboratory setting, customer anger in call centers is based on genuine concerns, e.g., receipt of poor products or service. A strong body of theoretical and empirical research suggests that communication of anger may be advantageous in confrontational situations, such as making complaints, because it lets the interlocutor know that there is a critical issue that must be addressed (Tamir, Mitchell, & Gross, 2008; Van Kleef, De Dreu, & Manstead, 2004). Thus, even if the state anger of callers who were played music with prosocial lyrics did dissipate while waiting for their calls to be answered, they may still have expressed anger outwardly to the employees who were rating customer anger. Alternatively, the duration of time that participants listened to the music to may not have been sufficient for the prosocial lyrics to take effect; while participants in Greitemeyer's (2011) laboratory studies listened to two full songs (Studies 2–5), callers in the field study listened to an average of just 75 seconds of music while waiting for their call to be answered. A further possibility is that cutting the music in the prosocial condition to fit a 2 minute loop of three songs could have influenced callers' perceptions of how long they had been on hold, such that they felt that they had been on hold for longer, leading to frustration (Unzicker, 1999). However, none of these explanations account for the finding that music with neutral lyrics did have positive effects when compared with the instrumental music.

Perhaps the most likely explanation for why the prosocial lyric effect does not generalize to call centers—and why music with neutral lyrics does reduce caller anger and employee exhaustion in this context—concerns the situationally dependent nature of people's experiences of music. Prior evidence suggests that the experience of music as a pleasant stimulus can reduce aggressive interpersonal behavior (Krahé & Bieneck, 2012), and that people are even willing to wait longer on hold to call centers when they are played music that they like (North et al., 1999). Yet the experience of music is not fixed; it is a product of the interaction between individuals and situations (Ruud, 2010). Although Greitemeyer's (2009a) piloting work identified no differences in liking between the music pieces with prosocial and neutral lyrics used in the present research, it was conducted in a controlled laboratory context in which participants were likely to feel neutral prior to listening to music. In call centers, the purpose of many calls is tocomplain about a product or service, meaning that customers may begin their call angry (Grandey et al., 2004); here, being played music with messages about “healing the world” and helping other people might act as an irritant. Indeed, research has suggested that people are disinclined to listen to songs that mismatch their moods. For example, Friedman, Gordis, and Förster (2012) showed that individuals in sad moods were strongly averse to listening to happy songs, because such songs would feel inappropriate. Similarly, when approaching confrontational situations where anger is a likely useful emotion, individuals have a preference to avoid music that will increase pleasant emotion (e.g., excitement), and instead choose to listen to music that will increase their anger (Tamir et al., 2008). Thus, music with prosocial lyrics may fail to reduce callers' anger in the call center context. In contrast, compared with instrumental background music, which callers may associate with negative constructs, such as complaining and waiting, due to its prevalence in call centers (Blair & Shimp, 1992), popular music with neutral lyrics that do not clash with one's current mood could provide a preferential alternative.

Although the present findings are suggestive of notion that people's experiences of music are situationally dependent, it should be noted that there are prior studies wherein the effects of music on interpersonal behavior have been translated from the laboratory to the field. For example, North et al.'s (2004) study of gym members' helping behavior found similar effects to those reported in the laboratory. Further research is therefore needed to test this explanation more directly, e.g., by contrasting songs with prosocial, neutral, and no lyrics in a laboratory-based call center simulator study, and manipulating callers' moods to be angry versus neutral prior to listening to the music. The prosocial lyric effect could also be tested within a different field context in which people are less likely to be angry prior to hearing the music, in order to test whether the effect does generalize to the field when the lyrics do not clash with the listener's current mood. One potential setting might be music venues, at the end of concerts. In such settings, the large number of people exiting the venue, combined with alcohol consumption, can lead to aggressive behavior, meaning that playing music with prosocial lyrics could serve to reduce aggression. However, unlike call centers, people usually experience elation rather than anger after a concert, and so music with prosocial lyrics would be unlikely to mismatch current mood.

A potential limitation of the research is that whereas laboratory conditions are highly controlled, in call centers there are many other events that can influence caller anger. In the present study, many of the factors that might introduce variance were accounted for in the design of the study (e.g., controlling for call waiting times, counterbalancing the intervention with all conditions occurring on each day of the week). However, potential confounds remained. For instance, it was not possible to control for events that might have influenced caller anger on a particular day during the study (e.g., if there was a website crash of one of the call center's client companies). Likewise, individual differences in employees' ability to deal with angry customers or their sensitivity to caller anger may not have balanced out if their shift patterns were irregular (potentially leading to more days in a particular condition). A second possible limitation regards the measurement of caller anger. Although recall biases were reduced with the use of measures taken at the end of each working hour (Hershcovis & Reich, 2013), biases could still have occurred, with employees' ratings, for example, being more influenced by their most recent caller. However, there is no clear evidence that caller anger systematically varies by time of the hour, meaning that calls received at the end of each hour could be taken as a representative sample from across the day. Moreover, the consistent pattern for both anger and emotional exhaustion (which was measured using a different time referent) suggests that the findings observed are unlikely to be the result of bias in the measurement of anger. Nevertheless, the results of the present research must be treated cautiously and replication is necessary before music with neutral lyrics can be recommended as an intervention for customer service call centers.

In conclusion, customer aggression is a highly prevalent phenomenon, with serious implications for employees' well-being and performance (Hershcovis & Barling, 2010), and interventions to prevent it are much needed. Although music with prosocial lyrics has been shown to be effective in prompting lower levels of aggression in laboratory conditions (Greitemeyer, 2011), the results of the present study suggest that these effects may not generalize to customer aggression in call centers. Instead, popular music with neutral lyrics emerged as a more promising candidate for interventions to reduce caller anger and improve employee well-being. Thus, while music with prosocial lyrics may not be able to heal the working world, music may still have an important role to play.
